# Infrared thermography unveiled the variation of brown adipose tissue thermogenesis among East Asian adults

**DOI:** 10.14814/phy2.70279

**Published:** 2025-03-20

**Authors:** Yuka Ishida, Kazuhiro Nakayama

**Affiliations:** ^1^ Department of Integrated Biosciences, Graduate School of Frontier Sciences The University of Tokyo Kashiwa Chiba Japan

**Keywords:** brown adipose tissues, cold tolerance, East Asian, infrared thermography

## Abstract

The thermogenesis of brown adipose tissue (BAT) is interesting because the contribution to human adaptation to cold and obesity resistance has been suggested. ^18^F‐fluorodeoxyglucose—positron emission tomography/computed tomography (FDG‐PET/CT) is a common method for measuring BAT activity; however, it has been studied in few large cohorts due to concerns about safety and cost. Studies using alternative methods make it challenging to directly compare BAT activity among studies and interpret those results because the procedure is various. We measured the supraclavicular BAT thermogenesis of 122 healthy Japanese and Chinese adults under mild cold stress using standardized infrared thermography (IRT) and examined the effects of various factors on BAT variation. BAT thermogenesis was significantly higher in females than in males (*p* < 0.001) and significantly higher in Chinese than in Japanese individuals (*p* < 0.05). Among the 27 participants enrolled in both summer and winter experiments, BAT thermogenesis increased during winter (*p* < 0.05) only in Japanese participants. Additionally, individuals born at higher latitudes exhibited greater BAT thermogenesis (*p* < 0.05), suggesting the involvement of genetic background or cold exposure in early life stages. We obtained interesting anthropological and physiological findings with the use of non‐invasive IRT.

## 1 INTRODUCTION

Long‐term energy imbalance causes metabolic diseases, such as obesity and type 2 diabetes (Hill et al., 2012). A better understanding of the factors that impact energy balance is critical for practical medical studies and health maintenance. As the incidence of obesity continues to increase worldwide, attention is being paid to brown adipose tissue (BAT) for its unique functions (Tam et al., 2012). Unlike white adipose tissue (WAT), which stores energy, BAT dissipates energy by producing heat and plays a role in maintaining core body temperature and energy homeostasis (Tam et al., 2012). This function of BAT is helpful for adaptation to cold environments, facilitating non‐shivering thermogenesis and increasing energy expenditure (Levy & Leonard, 2022; Poekes et al., 2015; Sazzini et al., 2014). As shown in the Sholander model, humans require additional energy expenditure when the ambient temperature falls below their thermoneutral zone, around 22–29°C (Brychta et al., 2024; Scholander et al., 1950). This means that physiological responses such as thermogenesis to maintain body temperature are important in cold environments. Understanding the dynamics of BAT and its role in human thermoregulation could have implications not only for managing metabolic diseases but also for exploring how humans have adapted to cold environmental conditions.

Cold exposure activates BAT via the sympathetic nervous system (Fenzl & Kiefer, 2014). Brown adipocytes contribute to non‐shivering thermogenesis in which oxidative phosphorylation is uncoupled from ATP synthesis. This process is mediated by uncoupling protein 1 (UCP1), which dissipates the proton gradient across the mitochondrial membrane and releases energy in the form of heat rather than ATP (Fenzl & Kiefer, 2014). Typically, BAT activity is assessed after mild cold exposure using sophisticated imaging techniques, such as ^18^F‐fluorodeoxyglucose—positron emission tomography/computed tomography (FDG‐PET/CT), which measures tissue glucose uptake (Saito et al., 2009). In studies of Japanese participants assessed using FDG‐PET/CT, a decrease in BAT activity with age was observed (Yoneshiro, Aita, Matsushita, Okamatsu‐Ogura, et al., 2011), and tests on the same individuals showed increased activity in winter compared to summer (Saito et al., 2009; Yoneshiro, Aita, et al., 2013; Yoneshiro et al., 2016). Additionally, among participants in their 20s, only about half had high BAT activities, with variation observed among participants of similar ages (Yoneshiro, Aita, Matsushita, Okamatsu‐Ogura, et al., 2011). In contrast, in a young European population of South Asian ancestry, almost all participants had higher BAT activity (Admiraal et al., 2013; Bakker et al., 2014). Genetic factors are involved in determining BAT activity. Allele combinations of single nucleotide polymorphisms (SNPs) in the UCP1 gene and β3 adrenergic receptor (β3–AR) gene showed an association with age‐related declines in BAT activity in the Japanese population (Yoneshiro, Ogawa, et al., 2013). In addition, SNPs in the β2 adrenergic receptor (β2–AR) gene were associated with variation in BAT activity in East Asians (Ishida, Matsushita, Yoneshiro, Saito, Fuse, et al., 2024).

Concerns regarding the invasiveness of radiation exposure and the high cost of FDG‐PET/CT have prompted researchers to explore alternative methodologies for evaluating BAT activity (Chondronikola et al., 2018). Some FDG‐PET/CT studies have reported BAT activity data assessed at thermoneutral temperatures in clinical populations (Becher et al., 2021; Cypess et al., 2009; Pfannenberg et al., 2010). However, a non‐cold exposure test cannot effectively assess activated BAT, making it challenging to compare findings. With the growing interest in the role of BAT in human physiology and evolution, the necessity for an alternative method that is reliable and applicable for large‐scale screening of BAT activity in healthy individuals is rising.

Several non‐invasive techniques have emerged as potential substitutes for FDG‐PET/CT, including near‐infrared time‐resolved spectroscopy (NIR_TRS_) (Hamaoka et al., 2020; Nirengi et al., 2016), magnetic resonance imaging (MRI) (Cai et al., 2024), and infrared thermography (IRT) (Jang et al., 2014; Nirengi et al., 2019). Of these alternative methods, IRT, which can measure BAT thermogenesis, is the least invasive and least expensive, making it suitable for inferring BAT activity in many individuals over multiple tests (da Rosa et al., 2023; Jang et al., 2014; Nirengi et al., 2019). For IRT, various cooling protocols have been used, such as hand immersion in cold water (El Hadi et al., 2016; Malpique et al., 2019), cooling blankets, and water‐perfused suits (Haq et al., 2017; Levy et al., 2021), in which the cooling temperatures and durations vary. Methodologies for assessing BAT thermogenesis using IRT also vary; some studies quantified BAT thermogenesis by subtracting the baseline supraclavicular temperature (*T*scv) from the post‐stimulation *T*scv (El Hadi et al., 2016; Haq et al., 2017), while others measure changes in the thermally active area (Malpique et al., 2019). A method has been proposed to estimate BAT thermogenesis by calculating the temperature difference (ΔTemp) between the supraclavicular fossa (Tscv), where BAT is present, and the chest (Tc), where it is absent, accounting for the effect of cold stimulation on skin temperature measurements (Jang et al., 2014; Nirengi et al., 2019). Despite being non‐invasive, cooling protocols and methodologies for assessing BAT thermogenesis vary widely and lack standardization in IRT studies (Jimenez‐Pavon et al., 2019). A few studies have reported variations in human BAT thermogenesis assessed under standardized conditions using IRT (Jimenez‐Pavon et al., 2019). While many studies have reported results for dozens of samples (Jang et al., 2014; Nirengi et al., 2019; Symonds et al., 2012), very few have included East Asian populations. As far as we know, no studies have evaluated seasonal differences in BAT thermogenesis within the same individuals using IRT. There is a limited understanding of the degree of BAT thermogenesis in healthy adults assessed using IRT and its potential impact on humans. This issue hinders the accurate interpretation of the differences in BAT activity among individuals and populations.

This study aimed to investigate individual differences in BAT activity using a standardized protocol to measure BAT heat production in the supraclavicular fossa. Our findings suggest that BAT thermogenesis in adults undergoes sex‐ and ethnicity‐specific seasonal changes. These results provide valuable insights into the factors influencing BAT function and highlight the importance of considering physical traits, sex, and ethnic background in BAT studies based on IRT.

## 2 MATERIALS AND METHODS

### 2.1 Experiment time

The experiments in this study were conducted during the summer (June–September) and winter (December–March) of 2021–2024. The monthly mean outdoor temperature in 2021–2024 was 24.8 ± 2.5°C in the summer and 6.2 ± 2.7°C in the winter (Japan Meteorological Agency, http://www.jma.go.jp/jma/menu/menureport.html). In both seasons, the participants participated in the experiment in the morning (9:00–12:00).

### 2.2 Participants

All data were collected at the University of Tokyo, Kashiwa, Chiba, Japan. The participants were recruited through a mailing list of the constituents of the Kashiwa campus at the University of Tokyo. Overall, 122 adults (male and female) participated in this experiment, with 61 and 34 individuals recorded only during winter and summer, respectively. The sample included 77 males and 45 females, comprising 74 Japanese and 48 Chinese individuals. Twenty‐seven of the 122 individuals participated in both winter and summer experiments. Ethnicity was divided into Japanese and Chinese based on self‐declarations. Some individuals overlapped with those in our previous studies (Ishida, Matsushita, Yoneshiro, Saito, Fuse, et al., 2024; Ishida, Matsushita, Yoneshiro, Saito, & Nakayama, 2024).

**FIGURE 1:**
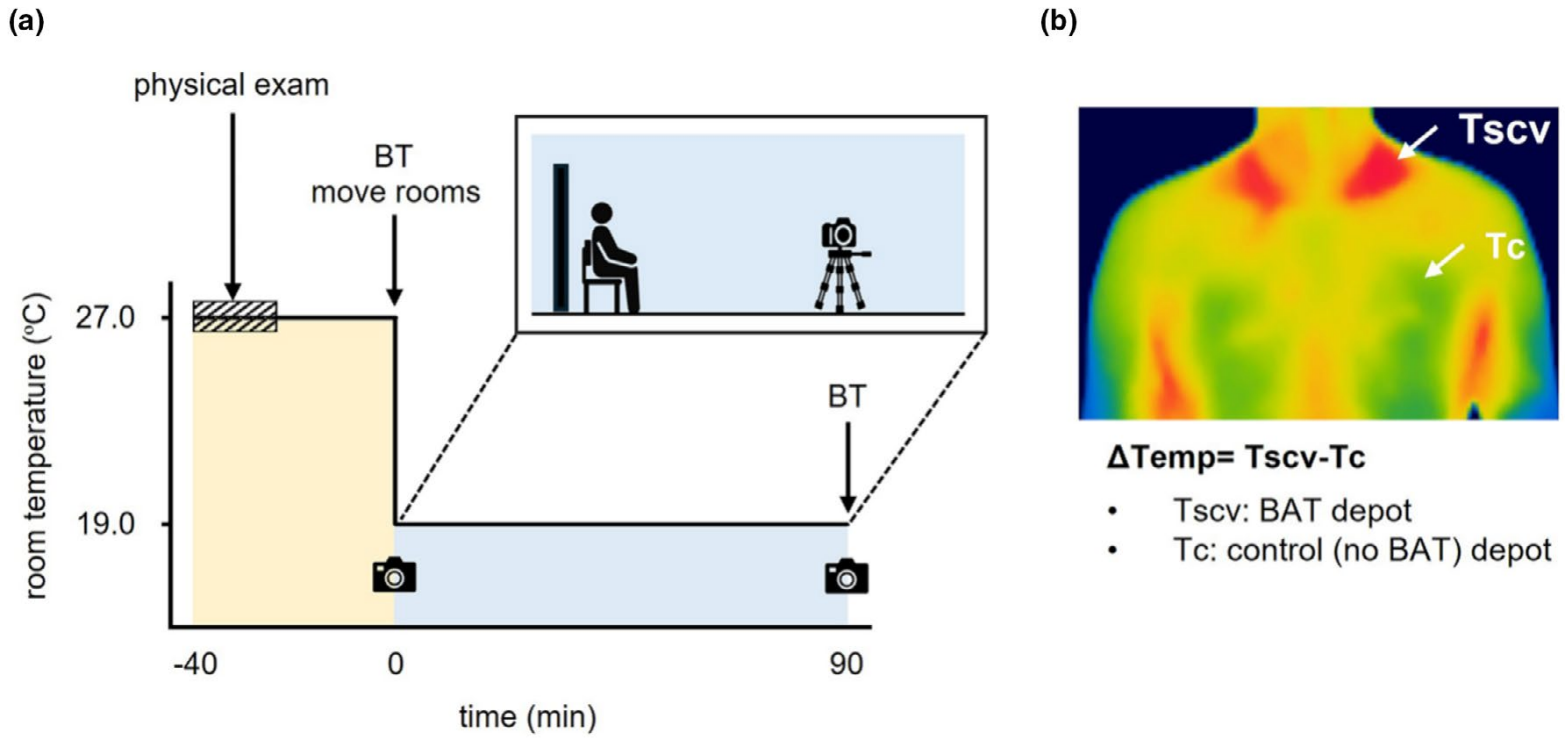
Details of the cold challenge. (a) The cold challenge protocol. Participants got a physical exam and rested at 27°C room (yellow fill) for 30 min. They were then moved to a room at 19°C room and were exposed to cold (blue fill) for 90 min. Time points captured the body surface temperature which was indicated in camera cartoons (after 0 and 90 min of cold exposure). The black panel behind the participants indicates a partition set for the background uniformity of the captured images. BT: Tympanic temperature measurements. (b) Infrared thermography (IRT) method for BAT thermogenesis determining based on body surface temperature. ΔTemp is regarded as the BAT thermogenesis of the IRT method and is calculated by the subtraction of the skin temperature of the control depot (*T*_c_) from the BAT depot (*T*scv).

The participants abstained from excessive drinking, staying up late, and exercising the previous day. On the day of the experiment, the participants skipped breakfast. They changed into a thin and lightweight disposable gown upon arriving at the laboratory and wore it throughout the entire period. First, they underwent standard physical examinations, including height, body weight, body mass index (BMI), muscle mass, body fat percentage, and waist circumference, in a 27°C room (Figure 1a). Height was measured using a stadiometer (Charder Electronic, Taichung, Taiwan). Body weight and body composition, including muscle mass and fat percentage, were measured using a dual‐frequency body composition monitor based on a bioelectrical impedance analysis (innerScan DUAL‐RD‐803L; TANITA, Tokyo, Japan). BMI was calculated as the body weight in kilograms divided by the square of height in meters. Waist circumference was measured using a tape measure.

### 2.3 Supraclavicular BAT thermogenesis measurements under the mild cold exposure

We performed cold exposure to stimulate the sympathetic nervous system and activate the BAT. Participants rested for 30 min in a room maintained at 27°C (mean ± SD: 26.8 ± 0.79°C) before transitioning to a 19°C (mean ± SD: 19.4 ± 0.50°C) room, where they were exposed to cold temperatures for 90 min (Figure 1a). This cold exposure condition was shown to effectively activate BAT without muscle shivering in Japanese adults (Saito et al., 2009; Yoneshiro, Aita, Matsushita, Kameya, et al., 2011; Yoneshiro, Aita, Matsushita, Okamatsu‐Ogura, et al., 2011). During this cold challenge, participants remained seated in a chair with a backrest, staying at rest throughout. In this study, we used the method previously proposed to estimate BAT thermogenesis by calculating the temperature difference (ΔTemp) between the supraclavicular fossa (Tscv), where BAT is present, and the thoracic region (Tc), where it is absent (Jang et al., 2014; Nirengi et al., 2019). The participants' left clavicle and control site were marked in advance using eyeliner as a reference for image analysis. The upper body surface temperature was captured using a FLIR‐E6‐XT thermal imaging camera (Teledyne FLIR, Wilsonville, Oregon, USA) at the start and endpoint of the cold challenge (Figure 1a) (Nirengi et al., 2019). The camera was positioned 0.7 m away from the participants and the emissivity was set to 0.98 as per the camera manual and previous study (Moreira et al., 2017). The camera was positioned perpendicular to the front of the participants and adjusted to neck height. FLIR Tools (Teledyne FLIR) were used to analyze body surface temperature. A circle was drawn covering each area, and the mean temperature of the circle was used to calculate the ΔTemp (Figure 1b) (Nirengi et al., 2019). In this paper, we refer to the cold‐induced thermogenesis of the supraclavicular fossa as “BAT thermogenesis.”

### 2.4 Core body temperature

Core body temperature was measured using the tympanic thermometer Kenon‐kun MC‐510 (Omron Healthcare, Kyoto, Japan). Rectal temperature measurement poses a burden to participants, and many were expected to hesitate to use this method. Instead, the tympanic temperature was chosen because it is well correlated with the rectal temperature (Duru et al., 2012) and closely approximates the nasopharyngeal temperature (Mangat et al., 2010). We measured the temperature in the right ear, and the same investigator conducted all measurements using a similar method.

### 2.5 Questionnaire investigation

Participants optionally provided their birthplace, their parents' birthplace, and their parents' residence during the conception month. Municipality information was converted to latitude values using Google Maps (https://www.google.co.jp/maps/) and analyzed for association between latitude and BAT thermogenesis. Geographic information was obtained as the latitude of the location of the municipal government building; if this information was unavailable, the latitude corresponding to the approximate center of the municipality was used. The estimated conception month was calculated as 9 months before the birth month. Participants answered questions about their sensitivity to heat or cold in daily life: 1 = sensitive to heat; 2 = neither; 3 = sensitive to cold. We evaluated the relationship between thermal comfort and the physiological data of individuals with thermal comfort (1 or 3 of three items).

### 2.6 Statistical analysis

The Kolmogorov–Smirnov test was conducted to assess the normality of the measured data, and appropriate statistical methods were selected accordingly. Correlations were assessed using Pearson's or Spearman's rank correlation coefficients. Analysis of covariance (ANCOVA) and Student's *t*‐tests were used to examine differences in BAT thermogenesis according to sex or ethnicity, while paired *t*‐tests were used to compare repeated measurement data. Multivariate analysis of variance (MANOVA) was used to assess differences by ethnicity and test season for participants who attended both the summer and winter tests (Figure 3a,b). The association between birthplace latitude and BAT thermogenesis was assessed using a multiple linear regression model. Differences in tympanic temperature changes and sensitivity to heat/cold were assessed using Student's *t*‐tests. Data are presented as mean ± SD. All statistical tests were two‐sided tests, and *p*‐values under 0.05 were considered statistically significant. Statistical analyses were performed using SPSS 27 software (IBM, Tokyo, Japan).

## 3 RESULTS

### 3.1 Participant's characteristics

The participants' characteristics are summarized in Table 1. None of the participants were extremely underweight or overweight. The differences in characteristics according to sex and ethnicity are shown in Tables 2 and 3, respectively. There were no differences in BMI between the sexes, but a higher body fat percentage was observed in females (Table 2). There was also no difference in BMI between the Japanese and Chinese participants, but the Chinese participants showed a higher body fat percentage (Table 3). The proportion of men was higher in the Japanese than in the Chinese population (Table 3). The characteristics of the participants who took part in both seasons are summarized in Table 4. Male participants who took part in both seasons showed no differences in body characteristics between ethnicities (Table 5). As only one Japanese female participant took part in both seasons, we did not compare the characteristics between female ethnicities. The characteristics of the Chinese female participants who took part in both seasons are shown in Table 6.

**TABLE 1:** Characteristics of all participants (*n* = 122).

*N* (male/female)	122 (77/45)
Age	26.9 ± 8.2
Height (cm)	168.8 ± 8.3
Body weight (kg)	61.7 ± 10.7
BMI (kg/m^2^)	21.6 ± 3.0
Muscle mass (kg)	44.4 ± 7.7
Body fat percentage (%)	23.6 ± 8.0
Waist circumference (cm)	76.4 ± 9.3
Ethnicity (Japanese/Chinese)	74/48

*Note*: Values are means ± SD.

**TABLE 2:** Differences in characteristics of sex.

	Males	Females	*p*‐value
*N* (Japanese/Chinese)	77 (53/24)	45 (21/24)	0.042^a^
Age	25.8 ± 6.4	28.7 ± 10.3	0.650^b^
Height (cm)	173.3 ± 5.8	161.2 ± 6.3	2.5E‐19^c^
Body weight (kg)	65.2 ± 10.1	55.7 ± 9.1	7.9E‐07^c^
BMI (kg/m^2^)	21.7 ± 3.2	21.4 ± 2.8	0.323^d^
Muscle mass (kg)	49.2 ± 4.9	36.1 ± 3.5	1.2E‐33^c^
Body fat percentage (%)	19.6 ± 6.3	30.5 ± 5.4	8.1E‐16^d^
Waist circumference (cm)	77.4 ± 9.6	74.8 ± 8.7	0.047^d^

*Note*: Values are means ± SD. *p*‐values were calculated using the following statistical methods.

^a^ Comparisons made by males versus females using Pearson's chi‐squared test.

^b^ Mann–Whitney *U*‐test.

^c^ Student's *t*‐test.

^d^ Multiple linear regression model adjusted by age.

**TABLE 3:** Differences in characteristics of Japanese and Chinese participants.

	Japanese	Chinese	*p*‐value
*N* (male/female)	74 (52/22)	48 (25/23)	0.042^a^
Age	27.7 ± 10.1	25.7 ± 3.0	0.225^b^
Height (cm)	168.9 ± 8.5	168.7 ± 8.2	0.025^c^
Body weight (kg)	60.6 ± 10.1	63.4 ± 11.5	0.005^c^
BMI (kg/m^2^)	21.2 ± 2.7	22.2 ± 3.4	0.043^c^
Muscle mass (kg)	45.0 ± 7.6	43.4 ± 7.8	0.104^c^
Body fat percentage (%)	21.3 ± 7.3	27.1 ± 7.7	0.001^c^
Waist circumference (cm)	75.1 ± 8.7	78.4 ± 9.9	0.021 ^c^

*Note*: Values are means ± SD. *P*‐values were calculated using the following statistical methods.

^a^ Comparisons made between Japanese and Chinese by Pearson's chi‐squared test.

^b^ Mann–Whitney *U*‐test.

^c^ ANCOVA controlled by sex.

**TABLE 4:** Characteristics of participants who joined in summer and winter experiments (*n* = 27).

*N* (male/female)	27 (18/9)
Age	28.0 ± 9.1
Height (cm)	169.1 ± 10.1
Body weight (kg)	63.8 ± 13.1
BMI (kg/m^2^)	22.3 ± 4.1
Muscle mass (kg)	44.6 ± 8.1
Body fat percentage (%)	25.4 ± 8.9
Waist circumference (cm)	78.6 ± 11.0
Ethnicity (Japanese/Chinese)	11/16

*Note*: Values are means ± SD.

**TABLE 5:** Differences in characteristics of Japanese male and Chinese male repeat participants (*n* = 18).

	Japanese male	Chinese male	*p*‐value
*N*	10	8	—
Age	28.0 ± 11.0	26.8 ± 3.1	0.256^a^
Height (cm)	172.6 ± 7.1	176.4 ± 6.8	0.261^b^
Body weight (kg)	64.3 ± 10.9	69.1 ± 13.5	0.416^b^
BMI (kg/m^2^)	21.6 ± 3.6	22.4 ± 5.3	0.682^c^
Muscle mass (kg)	47.6 ± 5.2	50.7 ± 4.4	0.191^b^
Body fat percentage (%)	21.1 ± 6.5	21.2 ± 8.2	0.926
Waist circumference (cm)	76.8 ± 10.4	82.2 ± 13.5	0.339^c^

*Note*: Values are means ± SD. *p*‐values were calculated using the following statistical methods.

^a^ Comparisons made by Japanese males versus Chinese males by Mann–Whitney *U*‐test.

^b^ Student's *t*‐test.

^c^ Multiple linear regression model adjusted by age.

**TABLE 6:** Characteristics of Chinese female repeat participants (*n* = 8).

Age	25.4 ± 2.3
Height (cm)	160.2 ± 6.1
Body weight (kg)	60.5 ± 13.8
BMI (kg/m^2^)	23.3 ± 3.7
Muscle mass (kg)	36.6 ± 5.3
Body fat percentage (%)	34.8 ± 5.4
Waist circumference (cm)	78.5 ± 10.0

*Note*: Values are means ± SD.

BAT thermogenesis showed a weak positive correlation with age in 122 participants (*p* = 0.031, *ρ* = 0.195). This correlation did not persist when the data was stratified by sex (males: *p* = 0.065, *ρ* = 0.211; females: *p* = 0.401, *ρ* = 0.128). No decline in BAT thermogenesis with age was observed, in contrast to previous studies showing age‐dependent decreases in BAT activity (Saito et al., 2009; Yoneshiro, Aita, Matsushita, Okamatsu‐Ogura, et al., 2011). This discrepancy is likely due to the fact that this study's participants did not include individuals older than middle age from the participant pool.

### 3.2 Sex and ethnicity differences in BAT thermogenesis

**FIGURE 2:**
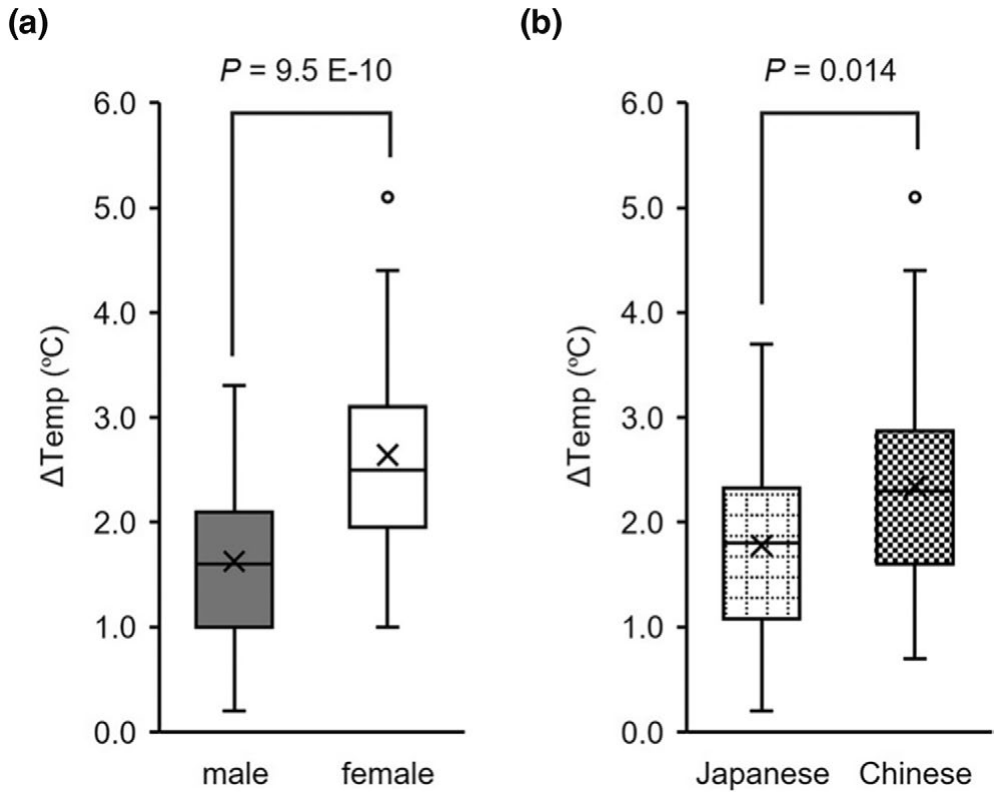
Differences in BAT thermogenesis by sex or ethnicity. (a) Sex differences in BAT thermogenesis; (b) Ethnicity differences in BAT thermogenesis; *P*‐values were calculated by (a) ANCOVA after controlling for sex, age, and test season. (b) ANCOVA after controlling for ethnicity, sex, age, and test season. The box plot shows median values (central line), mean values (cross mark), and 75th and 25th percentiles (upper and lower boundaries). The largest and smallest values are represented as whiskers drawn from the ends of the box to the values. Outliers are indicated as dots.

Female BAT thermogenesis was higher than that of males (*p* = 4.1E‐10, Cohen's *d* = −1.28, 2.64 ± 0.91°C vs. 1.63 ± 0.72°C, Student's *t*‐test). In a previous study, BAT activity was negatively correlated with age (Yoneshiro, Aita, Matsushita, Okamatsu‐Ogura, et al., 2011). Seasonal differences in BAT activity have also been reported in the Japanese population (Ishida, Matsushita, Yoneshiro, Saito, Fuse, et al., 2024; Saito et al., 2009; Yoneshiro et al., 2016). Thus, we controlled for these covariates using an ANCOVA. This significant finding persisted even after controlling for age and test season (Figure 2a, *p* = 9.5E‐10, partial *η* ^2^ = 0.273).

Japanese BAT thermogenesis was lower than that of the Chinese (*p* = 8.2E‐04, Cohen's *d* = −0.64, 1.78 ± 0.81°C versus 2.35 ± 1.00°C, Student's *t*‐test). As shown in Table 3, the male‐to‐female ratio differed according to ethnicity, which could also contribute to variations in BAT thermogenesis. We also conducted an ANCOVA controlling for age, test season, and sex to account for potential effects. Ethnic differences persisted even after the ANCOVA (Figure 2b, *p* = 0.014, partial *η* ^2^ = 0.051).

### 3.3 Seasonal variation of BAT thermogenesis

**FIGURE 3:**
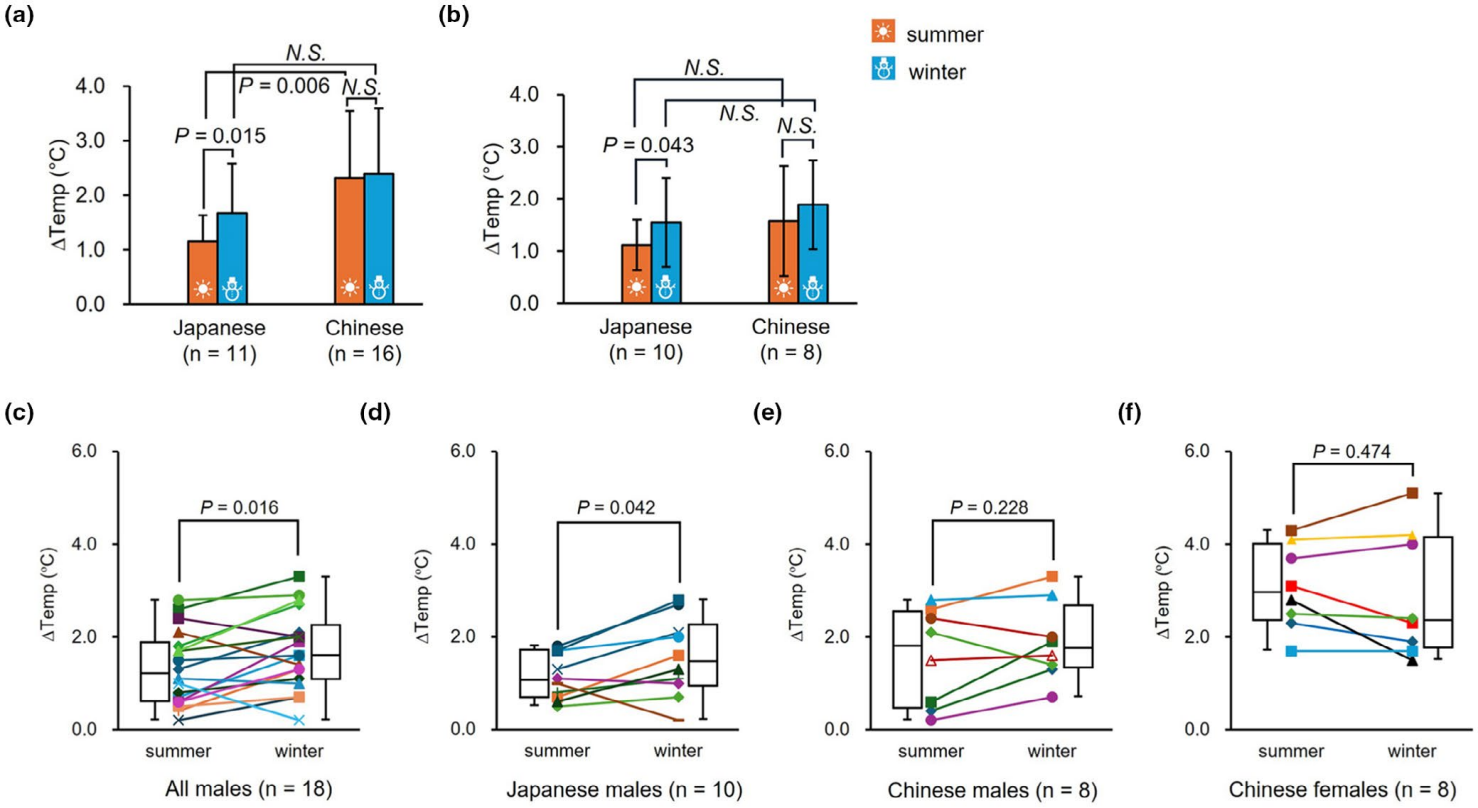
Seasonal changes in BAT thermogenesis according to ethnicity and sex. (a, b) Seasonal changes in BAT thermogenesis for participants who joined in two seasons are shown by ethnicity. (a) All repeat participants (*n* = 27), (b) male repeat participants (*n* = 18); *p*‐values were calculated by multivariate analysis of variance (MANOVA) and adjusted with Bonferroni post hoc correction. The difference color of the bars indicates the experiment season. Orange: Summer; Blue: Winter; (c–f) Seasonal changes in BAT thermogenesis for male participants who joined in two seasons (*n* = 18) are shown by sex. (c) Japanese and Chinese males (*n* = 18); (d) Japanese males (*n* = 10); (e) Chinese males (*n* = 8); (f) Chinese females (*n* = 8); We do not show the result of Japanese female because there was only one participant. The colored lines show seasonal changes in BAT thermogenesis for each participant. Box plots show median values (central line), mean values (cross mark), and 75th and 25th percentiles (upper and lower boundaries, respectively). The largest and smallest values are represented as whiskers drawn from the box ends. *p*‐values were calculated by paired *t*‐test. *N.S*. means *p* > 0.05. All data were presented at mean ± SD.

Twenty‐seven individuals who participated in both winter and summer experiments were tested using a paired *t*‐test to determine whether there were any seasonal differences in BAT thermogenesis. Among these, 10 of the 18 males and one of the nine females were Japanese (Table 4). Extremely underweight or overweight participants did not take part in this study. Japanese participants showed higher BAT thermogenesis in winter than in summer (*p* = 0.020, Cohen's *d* = −0.84, 1.67 ± 0.90°C vs. 1.16 ± 0.47°C), whereas no such seasonal change was observed in Chinese (*p* = 0.694, Cohen's *d* = −0.10, 2.39 ± 1.21°C vs. 2.32 ± 1.22°C). We performed analyses stratified by ethnicity and sex to explore these patterns. The results of the MANOVA revealed a significant seasonal change in BAT thermogenesis in the Japanese participants (Figure 3a, *p* = 0.015, partial *η* ^2^ = 0.21). When comparing ethnic groups within the same season, Japanese participants exhibited 1.16 ± 0.39°C lower BAT thermogenesis in the summer compared to Chinese participants (Figure 3a, *p* = 0.006, partial *η* ^2^ = 0.26), whereas winter BAT thermogenesis was similar in both groups (Figure 3a, *p* = 0.108, partial *η* ^2^ = 0.10). Since BAT thermogenesis was higher in females and the male/female ratio was different between the two ethnic groups, we analyzed the male subset of the 27 participants (Table 5). No significant difference in body composition was observed between Japanese and Chinese males (Table 5). We confirmed significant seasonal differences only in Japanese males but not in Chinese males (Figure 3b, *p* = 0.043, partial *η* ^2^ = 0.232), whereas the observed ethnic differences in summer were absent, and no significant ethnic variation was found in either season (Figure 3b, summer: *p* = 0.239, partial *η* ^2^ = 0.085, winter: *p* = 0.416, partial *η* ^2^ = 0.042).

Additionally, data from each individual were visualized to examine the diversity of seasonal changes according to nationality and sex (Figure 3c–3f). When analyzing all 18 males, BAT thermogenesis was higher in winter than in summer (Figure 3c, *p* = 0.016, Cohen's *d* = −0.63, 1.70 ± 0.84°C versus 1.32 ± 0.80°C). Japanese males exhibited significantly higher BAT thermogenesis in winter compared to summer (Figure 3d, *p* = 0.042, Cohen's *d* = −0.75, 1.55 ± 0.85°C vs. 1.12 ± 0.48°C). This seasonal change was not observed in Chinese males (Figure 3e, *p* = 0.228, Cohen's *d* = −0.47, 1.89 ± 0.85°C vs. 1.58 ± 1.05°C), as shown in the MANOVA. In the female analysis, Japanese females were excluded because of the small sample size (*n* = 1). Chinese female participants did not show significant seasonal differences (Figure 3f, *p* = 0.474, Cohen's *d* = 0.27, 2.89 ± 1.35°C vs. 3.06 ± 0.91°C).

Among participants who took part in the experiment across both seasons, differences in BAT thermogenesis between the seasons were not correlated with age (*n* = 27, *p* = 0.653, *ρ* = −0.091) and changes in core body temperature (*n* = 11, *p* = 0.638, *r* = 0.160).

### 3.4 Relationship between questionnaire information and BAT thermogenesis

**FIGURE 4:**
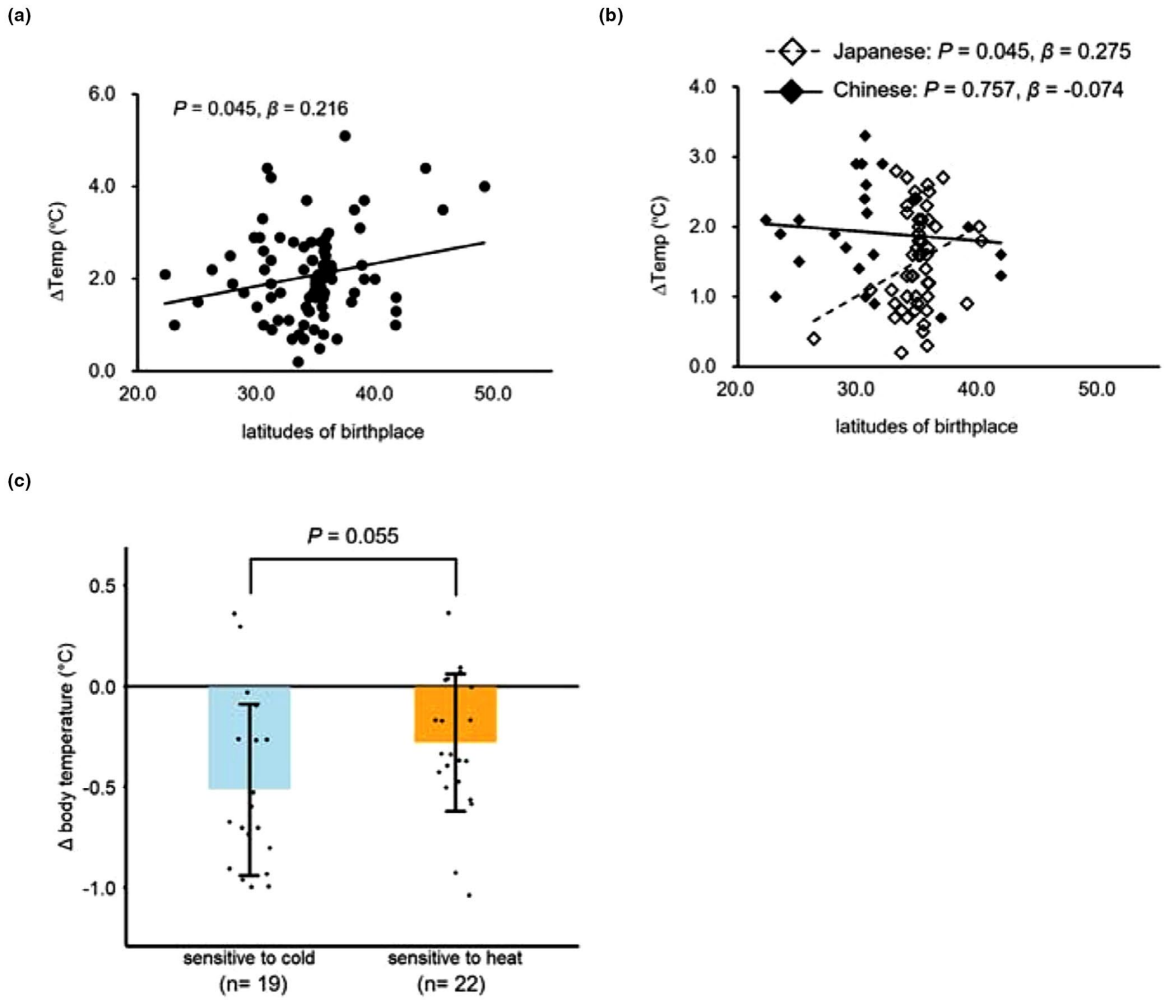
Questionnaire topics involved in the variation of BAT thermogenesis. Association of (a) BAT thermogenesis in winter and latitude of all participants' birthplaces (*n* = 87); (b) BAT thermogenesis and latitude of male birthplaces stratified by ethnicity (*n* = 76); (c) Variations in core body temperature changes in cold environments depending on sensitivity to heat or cold. The *n* in brackets indicates the number of participants. Black dots indicate individual data point. (a) *p*‐value and standardized *β* were calculated by linear regression model. (b) *p*‐value and standardized *β* were calculated by linear regression model adjusted by test season. (c) *p*‐value was calculated using the Student's *t*‐test. Data were presented at mean ± SD.

It has been suggested that cold exposure during juvenility contributes to higher BAT activity in adulthood (Levy et al., 2021), and that parental cold exposure before conception is involved in BAT activity in their offspring (Sun et al., 2018). Given that higher latitudes are correlated with lower temperatures, we assessed the correlation between BAT thermogenesis and latitude using a linear regression model with birthplace, parents' residence during the estimated conception month, and parents' birthplace as independent variables. The estimated conception month was calculated as 9 months preceding the birth month. Strong correlations were observed among these variables (*ρ* > 0.8, Spearman's rank correlation coefficient). Participants born at higher latitudes exhibited higher BAT thermogenesis during winter (Figure 4a, *p* = 0.045, *β* = 0.216). Since sex and ethnic differences in BAT thermogenesis were observed (Figure 2a,b), and all latitude values provided by participants were sex‐biased (*p* < 0.05, Mann–Whitney *U*‐test), we stratified the correlation analysis by sex and ethnicity. In Japanese males, a positive correlation was observed between birthplace and BAT thermogenesis (Figure 4b; *p* = 0.045, *β* = 0.275). The latitude of parental residence before conception was also positively correlated with BAT thermogenesis (*p* < 0.05). Birthplace latitudes of their fathers showed marginal positive correlations (*p* = 0.067, *β* = 0.276), and those of their mothers showed significant positive correlations (*p* = 0.018, *β* = 0.355). However, these positive correlations were not observed among Chinese males.

BAT thermogenesis did not differ by self‐reported heat/cold sensitivity (*p* = 0.535, Cohen's *d* = −0.13, 1.90 ± 0.83°C vs. 2.02 ± 0.93°C). However, participants who answered with greater sensitivity to cold in their daily lives tended to have a decrease in core body temperature during cold exposure compared to those who answered being more sensitive to heat (Figure 4c, *p* = 0.055, Cohen's *d* = 0.619, − 0.52 ± 0.43°C vs. −0.28 ± 0.33°C).

## 4 DISCUSSION

In this study, we present findings on the diversity in BAT thermogenesis related to sex and ethnicity using a standardized BAT measurement method. Furthermore, we address the concerns highlighted in this report regarding the assessment of BAT using IRT. Most previous human adult BAT studies were retrospective, often relying on FDG‐PET/CT conducted for clinical purposes such as cancer staging, and typically lacked cold stimulation or a standardized methodology (Becher et al., 2021; Fletcher et al., 2020; Fuller‐Jackson et al., 2020; Herz et al., 2021; Pfannenberg et al., 2010). This issue has hindered the comparison of significant findings. An important aspect of this study is the use of a standardized IRT methodology for all 122 participants across different ethnicities and sexes. BAT thermogenesis was significantly higher in females than in males, and significantly higher in Chinese than in Japanese individuals. Among the 27 participants enrolled in both summer and winter experiments, BAT thermogenesis increased in Japanese participants during winter, whereas Chinese participants exhibited consistently high activity regardless of the season. Additionally, individuals born at higher latitudes exhibited greater BAT thermogenesis.

### 4.1 Sex differences in BAT thermogenesis were observed in cold‐stimulated IRT

Previous studies have reported higher BAT activity in females than in males; however, this observed sex difference remains unclear due to inconsistencies in study design, particularly in methods of sympathetic stimulation via cold exposure (Becher et al., 2021; Fletcher et al., 2020; Fuller‐Jackson et al., 2020; Herz et al., 2021; Pfannenberg et al., 2010). We assessed BAT thermogenesis using IRT after cold exposure in all participants and confirmed that BAT thermogenesis was more active in females than in males (Figure 2a).

Molecular studies in rodents support the higher BAT activity in females. Compared to male rat BAT, female rat BAT contains higher levels of UCP1, more multilocular lipid droplets, and a greater abundance of mitochondria, indicating an enhanced capacity for BAT thermogenesis (Rodríguez‐Cuenca et al., 2002). Surgical removal of ovaries reduced *Ucp1* mRNA expression in BAT (Pedersen et al., 2001), whereas estradiol administration induces its expression in BAT (de Martínez Morentin et al., 2014). Estradiol directly activates BAT and enhances its activity via the hypothalamic neuronal circuit (Xu & López, 2018). Our observation that females have higher BAT thermogenesis may reflect these underlying mechanisms.

However, some studies have questioned whether sex differences in human BAT activity are influenced by cold stimulation. The general belief that females feel colder at higher ambient temperatures than males is often attributed to differences in body size and basal metabolic rate (Kingma et al., 2012; Parkinson et al., 2021); however, few studies have focused on BAT thermogenesis. A study based on the Scholander model found that young lean females required heat production at lower temperatures than young lean males (mean ± SD: 21.9 ± 1.3 vs. 22.9 ± 1.2°C) (Brychta et al., 2024). However, this sex difference was not linked to variations in BAT activity measured by FDG‐PET/CT and may be explained by greater insulation from body fat, as females typically have a higher body fat percentage (Brychta et al., 2024). Further investigations using physiological data from larger and more diverse populations are needed to determine sex differences in maintaining body temperature through BAT thermogenesis.

### 4.2 Seasonal variation of BAT thermogenesis is more prominent in Japanese than in Chinese

Cold‐adapted human populations exhibited considerable variations in BAT activity when using the same BAT evaluation method. Indigenous Siberians exhibited higher BAT activity than residents of the USA (Duru et al., 2012). The results of this study are valuable as previous studies did not compare BAT thermogenesis during the same season or conduct repeated measurements between populations. BAT thermogenesis remained relatively constant between summer and winter in the Chinese participants (Figure 3a,e,f), but Japanese participants exhibited clear seasonal variation, with significantly higher BAT thermogenesis in winter (Figure 3a,d).

Furthermore, when comparing BAT thermogenesis between Japanese and Chinese participants in the same season, no differences were observed in winter; however, Japanese participants exhibited significantly lower BAT thermogenesis in summer than Chinese participants (Figure 3a). The observed differences between populations in this study may be attributed to genetic factors. Modern Japanese populations represent a genetic mixture of the Jomon and Yayoi people. This well‐established theory is referred to as the dual‐structure model. The Jomon people were hunter‐gatherers who inhabited Japan at least 12,000 years ago, while the Yayoi people were agriculturalists who migrated to Japan from North Asia around 2300 B.C. (Rasteiro & Chikhi, 2009). The Yayoi people are characterized by larger body size and shorter limb proportions, which are speculated to be more cold‐adaptive than the morphology of the Jomon people (Seguchi et al., 2017). A recent genetic study indicated that the present‐day local Japanese populations with a higher ancestry proportion of the Jomon people tend to be overweight and have higher blood triglyceride and glucose levels (Watanabe & Ohashi, 2023). This suggests that the Jomon people might have evolved energy‐saving traits to cope with starvation (Watanabe & Ohashi, 2023). Higher BAT activity is positively correlated with higher energy expenditure (Ishida, Matsushita, Yoneshiro, Saito, Fuse, et al., 2024; Yoneshiro, Aita, Matsushita, Kameya, et al., 2011). Thus, the Japanese energy‐saving trait derived from the Jomon lineage may explain the significant decline in BAT thermogenesis during summer, when the degree of thermogenesis is less required than in winter.

Alternatively, ethnic differences in BAT thermogenesis may be attributed to body fat percentage, which could contribute to insulation (Wijers et al., 2010). However, the Japanese body fat percentage was lower than that of the Chinese, even after controlling for sex (Table 3). Thus, body fat insulation is unlikely to affect BAT thermogenesis in Japanese participants. This result, which showed lower BAT thermogenesis in summer than in winter, suggests that Japanese individuals may be more responsive to cold exposure, relying on increased non‐shivering thermogenesis to maintain body temperature during colder months. These findings suggest that different ethnic groups exhibit distinct physiological adaptations to cold environments. However, BAT thermogenesis could not be compared specifically for women, as there was only one Japanese female participant who attended both seasonal tests. A larger sample size is necessary to determine whether the seasonal variations observed in Japanese males are also present in Japanese females.

### 4.3 Geography may influence variations in BAT thermogenesis

Given that previous studies have suggested an involvement between childhood cold exposure and parental cold exposure history with variations in BAT activity (Levy et al., 2021; Sun et al., 2018), we surveyed participants' residential histories through a questionnaire. We then analyzed the correlation between latitudinal data and BAT thermogenesis. Individuals born at high latitudes tend to show higher BAT thermogenesis, likely due to greater exposure to cold stress during childhood. Birthplaces and childhood residences are assumed to be close, and those born at high latitudes are likely to experience more frequent cold exposure. The high frequency of cold exposure during early childhood affects BAT activity in adulthood (Levy et al., 2021), suggesting that the higher BAT thermogenesis observed in our participants from higher latitudes may have been influenced by temperature. Furthermore, in Japanese males, the estimated latitude of the parents' residence before conception positively correlated with higher BAT thermogenesis. Fathers' birthplaces were marginally correlated with participants' BAT thermogenesis, suggesting that fathers' histories of cold exposure might have intergenerational effects on their offspring. This result matched that of the offspring of mice exposed to cold before conception, which exhibited increased BAT activity due to epigenomic changes (Sun et al., 2018). Since mitochondrial DNA (mtDNA) is maternally inherited, the offspring's mtDNA represents their maternal genetic background. The positive correlation between maternal birthplace and BAT thermogenesis suggests the impact of mtDNA because seasonal cold adaptability could be explained by the mtDNA haplogroup (Nishimura et al., 2012). Given the degree of admixture between the Jomon and Yayoi populations varies across regional populations in Japan, these correlations with geographic information may be explained by the genetic diversity involved in the formation of the Japanese population. In addition, we should note that the participants self‐reported the parents' residences; thus, their accuracy could not be determined. Further studies should be conducted to investigate detailed cold exposure experiences to understand the impact of generations on human BAT thermogenesis, including cultural factors such as variations in parental living environments and the frequency of outdoor activities during childhood (Levy et al., 2021).

Although cold sensitivity was not significantly associated with BAT thermogenesis, individuals more sensitive to cold tended to have lower body temperatures with cold exposure. The involvement of BAT in maintaining core body temperature needs to be studied with a larger sample size while controlling for differences in insulation and basal metabolic rates.

### 4.4 Limitations

First, the BAT thermogenesis was evaluated using only ΔTemp. The record of changes in energy expenditure due to cold exposure provides stronger evidence of BAT activation (Yoneshiro, Aita, Matsushita, Kameya, et al., 2011; Yoneshiro et al., 2016). Second, ΔTemp is the subtraction of the skin surface temperature of Tc from the Tscv (Nirengi et al., 2019). Body segments with high‐fat percentages tend to have lower skin surface temperatures (Salamunes et al., 2017). Individuals with a large amount of chest fat who are severely obese or those with a small amount of chest fat who are skinny tend to exhibit lower or higher chest skin surface temperatures than individuals of normal weight and can falsely assess BAT thermogenesis. Chest skin fat thickness varies among individuals, and the data should be interpreted accordingly. Third, the menstrual cycles were not unified. Since the basal body temperature of females fluctuates with their menstrual cycle (Fuller‐Jackson et al., 2020) and hormones affect BAT activity (Fuller‐Jackson et al., 2020; Xu & López, 2018), these variations may influence IRT by measuring the body surface temperature. This study could not address whether this cycle contributes to the variation in BAT thermogenesis or seasonal changes among female participants due to incomplete data on hormone replacement therapy or menstrual cycles. Fourth, external stimuli that activate BAT include cold exposure and dietary components such as tea catechins and capsaicin (Yoneshiro et al., 2012; Yoneshiro et al., 2017). Therefore, when assessing BAT thermogenesis, it is important to consider participants' frequency of exposure to chronic cold stimuli (e.g., swimming or triathlons), their diets, or standardize their backgrounds. Fifth, since IRT does not directly measure BAT activity, an FDG‐PET/CT test for selected participants would be required to strengthen our findings. Finally, our investigation was limited to East Asians from Japan and China. Comparisons should be made using various populations with different genetic backgrounds or lifestyles to further elucidate variations in BAT.

### 4.5 Conclusions

This study aimed to understand the diversity in human BAT thermogenesis by employing a standardized IRT approach. We found that BAT thermogenesis differed according to sex and ethnicity. Furthermore, Japanese BAT thermogenesis in summer was weaker than that of the Chinese population. People who grew up at high latitudes tended to have higher BAT thermogenesis as adults, suggesting that early exposure to cold may play a role in the development of adaptive thermogenesis. The subjective perception of cold may be closely linked to the ability to maintain body temperature through BAT activation. These results will contribute to clarifying the connection between BAT thermogenesis and metabolic abnormalities and also provide valuable clues for understanding the process of adaptation to cold environments in humans.

## AUTHOR CONTRIBUTIONS

Y.I. and K.N. conceived and designed the research; Y.I. performed the experiments and data analysis; Y.I. and K.N. interpreted the results of the experiments; Y.I. prepared the figures; Y.I. drafted the manuscript; Y.I. and K.N. edited and revised the manuscript; and Y.I. and K.N. approved the final version of the manuscript.

## FUNDING INFORMATION

This work was supported by JSPS KAKENHI grant numbers JP21H02571, JP24K02106 (to Kazuhiro Nakayama), and JP23KJ0705 (to Yuka Ishida).

## CONFLICT OF INTEREST STATEMENT

The authors declared no conflicts of interest, financial or otherwise.

## ETHICS STATEMENT

The Ethics Committee of the University of Tokyo approved this study (21‐6), and the study design complied with the principles of the Declaration of Helsinki. All the participants provided written informed consent before inclusion in the experiments.

## Data Availability

Data will be made available upon reasonable request.
